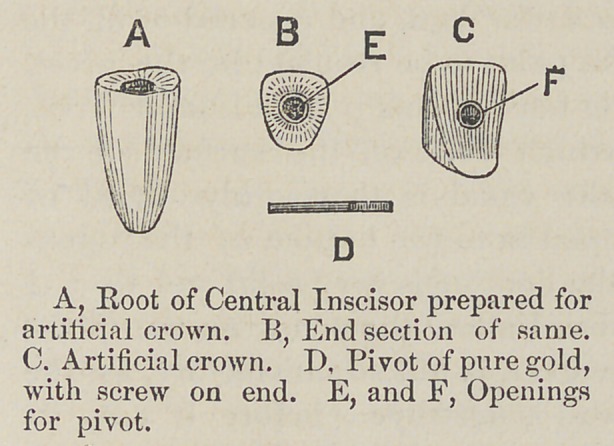# A New Style of Pivot Teeth

**Published:** 1874-09

**Authors:** W. H. Shadoan


### DENTAL REGISTER.

VOL. XXVIII.] SEPTEMBER, 1874. [NO. 9.

### A NEW STYLE OF PIVOT TEETH.

BY W. H. SHADOAN, D. D. S.

*Read before the District Dental Society of the Seventh Judicial District of the State of New York. Rochester, N. Y., June 2d. and 3d.*, 1874.

*Mr. President, and Gentlemen:*—I propose briefly to call your attention to a branch of operative dentistry (pivot teeth) that for the last few years has been grossly neglected. Especially is this true since the introduction of the abomination of abominations, Rubber, as a base for artificial dentures. I have no doubt that to many this will seem an old and antiquated subject for this paper; and so it is, and that in part is just why I have selected it.

How many of you present to-day have not known good, strong, healthy roots of teeth, extracted to make room for a rubber plate—teeth that ought never to have been extracted under any pretense whatever—by that class of men the introduction of rubber has brought into the profession.

You all know full well that there are more incompetent men in the dental profession to-day, than ever before; and you all know where to attach the blame. You in New York State may be free from this class of men; but out West, there are men who make it an every day business of extracting all the teeth that they can lay their hands on; and then supply the place with a plate. These men extract many thousands of teeth that in skillful hands could easily be preserved for years of usefulness and comfort by filling. But there are teeth often presented that the ravages of decay have placed beyond repair, removing almost or quite the entire crown, and yet the root is healthy and strong. I have many of this class presented, some persons have a gold crown built on, but others refuse on account of the appearance of the gold. To extract such teeth is, I consider, a great wrong. Then to meet these demands, and at the same time preserve the roots of these teeth in their sockets—a matter of great importance in many cases—recourse must be had to other methods; and here to succeed, first class operations are demanded. No slip-shod, half way operations will stand the test or meet the demands of the profession any better in pivoting than in filling teeth The same laws of health hold good in pivot teeth as in filling; the health of the patient, the character of the teeth, as well as the secretions of the mouth, all go far toward modifying the permanency of the operation.

As a profession, we want first of all, first class operators and first class instruments, and appliances to facilitate the operations; and at the same time we must not ignore old and well tried principles; we should study them well and develop principles, using discrimination so as to be able to comprehend the complications present, and be able to meet every case presented to us with a due sense of the responsibility which should characterize the members of our profession; without all which injury must result to the patient and the operator.

Our profession should be a constant field of research for the truth, and when obtained and applied, we are all benefited thereby. But I must not forget that I promised to say something about pivot teeth. and before going into the details of the operation, I propose to say a few words in vindication of pivot teeth.

It is a well known fact that, the old way of doing things is not always the best way, but if the old way has met with success it then is entitled to consideration. It is sometimes argued that pivot teeth don't last long, and that after they fail the root is "so hard to get out." This is a very flimsy excuse. Sometimes a first class filling does not last long, but does it follow that we are not to fill teeth because a failure sometimes occurs? I have a tooth in my possession that had been pivoted twenty-three years, and when I attempted to take away the crown preparatory to removing the root to make room for a full upper set, I found the crown so firmly set that the whole was extracted together. How much better did the adjoining remain that were sound at the time? A Mrs. C., of Cleveland, Ohio, had worn one for twenty-two years; a Mr. C., of Kentucky, wore one for twenty years; a Mr. M., of Kentucky, has one that has been in for twenty-six years and has never had anything done to it. This reference to cases could be extended indefinitely, but the point is to show that the old method of pivoting teeth was not a failure by any means. But what I wish to call your attention to more particularly is a new style of pivot teeth. It may not be new to you, but it is comparatively new in this locality, and I assure you that, to my mind, it excels all methods that I am acquainted with. It has many advantages over all other kinds of work that I have ever tried or seen.

One reason why pivot teeth as a general rule last so short a time, is the careless, indifferent manner in which they are inserted. Many dentists tell their patients that pivot teeth are of very little account, and last but a little while, and then go to work to make good their assertion. No wonder that failure follows such operations. The man who is whipped before he goes into battle never wins a victory. So with the dentist; he who looks for failure in his operations, seldom meets with anything beyond his expectations. I believe in, and practice, as thorough work in pivot teeth as in other dental operations. This principle should never be deviated from, no matter what the circumstances may be. Pivot teeth, like amalgam fillings, as a general rule are not in very good repute, and when they are resorted to, it is on the principle that they are only temporary, and hence labor bestowed on them is labor lost. This is a bad principle; anything that is worth doing at all, is worth doing well.

My own practice is to save the tooth without pivoting if possible; sometimes, however, I deem it possible and even desirable to save the tooth by restoring the lost portion with gold; but I am not always master of the situation, and then I have to do the next best thing which is to take off the remaining portion of the crown and insert one on a pivot.

I resort to the pivot more frequently now than a few years ago; in other words, extract fewer teeth now to make room for plates than ever before. Those of the ten anterior teeth that can be saved by filling, even if the greater part is gone, are preserved, and the balance that have healthy roots are pivoted. Where there is only an occasional one gone, should most or all of the ten teeth, be in the condition described, pivoting should not be employed.

It is hardly necessary to say anything on the old method of pivoting teeth, further than to state one or two points that I always observe in performing this operation. These points are, to fill the root as I would if the crown was still remaining and to be filled; first, pass a few fibres of fine cotton or flax saturated with carbolic acid up to the foramen and then mallet in the gold until the point is reached that the pivot is to touch. The next point is to make as perfect fit or joint between the crown and root, as is possible, and to use fine hickory wood soaked in a solution of carbolic acid or creosote. Another point is to file the root off above the margin of the gum far enough that when the gum heals after the operation it will close in around the crown, and thereby exclude particles of foreign substances from lodging between and around the end of the root. As to making an absolute fit between the crown and root, that is simply out of the question; and as for interposing any substance that will insure a perfect fit or joint, so far as I have been able to find, is hardly possible; but thanks to somebody, unknown to me, there is a way of forming an absolute protection to the root of the tooth, preventing future decay and loss of the tooth, just as surely as the filling of a tooth prevents further decay; and it is to bring this before you, that I write this paper.

A few years ago, I believe in 1872, Dr. F. Peabody, of Louisville, Kentucky, returned from South America, and while in the establishment of C. Ash & Sons, of London, he found a new kind of pivot tooth. He procured an assortment and brought them home with him. They are made around a platina tube in the right position for the pivot, the tube forming the hole extends through the tooth. This platina lining to the hole in the tooth is about the thickness of No. 22 or 23 American gague plate. The bicuspids are pivoted as well as the six anterior teeth, which increases their scope considerably. For the sake of convenience, I have had some cuts prepared to illustrate this kind of work.

A, Root of bicuspid tooth prepared for artificial crown. B, End section of bicuspid. C, Artificial crown of pivot tooth. D, Gold pivot for same. E, Opening in root for pivot. F, Hole in crown of tooth.

In an examination of these cuts it will be seen that Fig. I. represents a bicuspid; root, crown and pivot. The preperation of the root for the reception of the crown, is as that for any other pivot, and with a suitable drill the outer portion of the nerve canal is enlarged, giving the root the appearance of having two holes in its end about equidistant from the border of the outer portion of the tooth, these holes are extended fully two-thirds of the length of the root, the last third of the depth of the hole some smaller than the first portion. The intervening portion of the tooth is then chiseled out from between the holes, up, say, about two-thirds the distance of the first opening; this gives the root the appearance of the section of the bicuspid as seen at G. in Fig. I. This opening should be somewhat larger than the pivot, so as to allow the filling to be well packed around it, and it should be flared at the surface or countersunk, leaving the stub a little higher at the outer border than at the opening for the pivot. The pivot D. in Fig. I., should be tried in the tooth to see if it has sufficient room to work freely, so that in introducing it at the proper time it can be done with facility, otherwise a failure, to locate it right will cause a little trouble in removing the investment. The pivot must be made of pure gold wire and of the exact size to fit the hole in the crown, so close that it will slip easily, otherwise in riveting down the end after the crown is adjusted, it will spring and jar and possibly not rivet down closely. The cavity being prepared, the pivot fitted, and the tooth selected, ground and arranged to suit, the next step is to adjust the coffer dam. This, in most cases, is very difficult, but is absolutely necessary to insure success, as the least moisture is as fatal as in filling teeth with adhesive gold. I use adhesive gold in inserting pivot teeth the same as in filling ordinary cavities, and am just as particular to make perfect work. The borders of the base for a pivot tooth require to be as perfect as in a contour filling. I have had to cut away the gum to make room for the dam, but it can always be applied if the requisite skill and perseverance are used. The dam being securely adjusted, the first step is to dry out the cavity and close the apicial foramen with a few fibres of flax saturated in carbolic acid, or gold saturated in the same will do as well, and some prefer it, and the remainder of the nerve canal filled with gold to near the point where the prongs of the pivot will reach. To be sure that the filling has not been carried too far, the pivot may be tried in its place. A paste of oxychloride of zinc is mixed a little thinner than for ordinary use, and the cavity filled half-full, the pivot forced to its place in the paste, and the crown applied and held steadily in position until the oxychloride hardens sufficiently to hold the pivot firmly, when the crown may be removed, leaving the pivot standing in the exact position in which it must remain. This done, the excess of oxychloride is excavated down nearly to the cross of the fork and sufficiently to give firmness and solidity to the remainder of the filling. The remaining portion of the cavity is filled with gold the same as other cavities, and should be carried about a line beyond the articulating surface of the root, being careful to make the borders of the fillings as firm and solid as possible, for upon this the crown rests as well as the permanency of the operation. After the filling is completed, the borders of the gold are dressed up smoothly, as well as the base around the pivot, and the crown refitted, when the outer or protruding portion of the pivot is clipped off a little way from the tooth and the end riveted down on the tooth and nicely polished. Then the coffer dam may be removed and the patient dismissed.

A, Root of Central Inscisor prepared for artificial crown. B, End section of same. C, Artificial crown. D, Pivot of pure gold, with screw on end. E, and F, Openings for pivot.

I will only occupy your time with one more case, Fig. II, which is a central inscisor. The two cuts illustrate the only two classes of pivot teeth, the single or round and the flat or bifurcated teeth. By a reference to Fig. II, it will be seen we have a central incisor, root, crown, section of root, and pivot. The principal difference in this cut from that of Fig. I, is the pivot and the preperation of the root. One is bifurcated and the other round, and these two cover the whole range of this class of pivot teeth. The operation of mounting a tooth on a pivot in the six anterior teeth is so nearly the same, that we will let the description of the central inscisor suffice for the whole.

In preparing one of these roots, the first thing after removing the old crown, is to use a burr drill nearly as large as the diameter of the root and drill down to the level of the gum. I find that by removing all except a thin portion of the outer part of the root, that the filling and leveling is less tedious as well as less painful; (these are all of importance, to to the patient at least.) Then dress down the end to the desired point and with a burr about as large, or nearly so, as the root, countersink the canal until the cut of the drill reaches the outer surface of the root, and with a file or a burr in a burring engine continue to slope to the more distant parts of the surface. Then with a burr of suitable size to make a hole about a line in diameter larger than the size of the pivot wire, the canal is opened from a sixteenth to an eighth of an inch, owing to the length of the root and size of the canal, then with a drill the size of the wire inside the thread. This allows the pivot to be tapped, which I do about the eighth of an inch; the balance of the canal is cleansed and filled the same as described.

Everything being now ready, select, grind and arrange the crown, and invest with the coffer dam, and proceed to fill the root as in other cases to the point to be reached by the pivot. If the gold is carried a little beyond this point it is immaterial, as the drill is used again, which levels off the surface of the root filling. This done, the canal is thoroughly dried of moisture and the pivot tapped in to the bottom of the opening for it. Sometimes if the dentine is very solid and firm, I use the screw tap, otherwise, I allow the screw on the pivot to form the tap. This, however, is not so satisfactory, as the gold is so soft that the thread is destroyed before it cuts its way. The better plan is to use the tap and thus insure the pivot reaching the bottom of the hole. Fill the remainder of the cavity and finish as in first description.

Now, gentlemen, I have hurriedly and imperfectly described a method of pivoting teeth that, if well and faithfully done, will be a great blessing to those who are so unfortunate as to need services of this kind. And I feel assured that your success and satisfaction will not be less than mine.

## Figures and Tables

**Figure f1:**
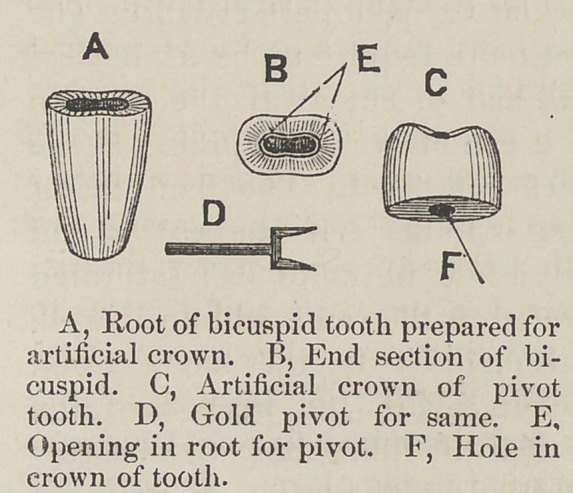


**Figure f2:**